# Supplementary material for: Comparative Efficacy of Animal Depression Models and Antidepressant Treatment: A Systematic Review and Meta-Analysis
Source: Pharmaceutics. 2024 Aug 29;16(9):1144. doi: 10.3390/pharmaceutics16091144 (PMC11435171; doi:10.3390/pharmaceutics16091144)
Supplement: Supplementary file 1 [file pharmaceutics-16-01144-s001.zip › supplement.pdf]

Table S1. MEDLINE via PubMed search result.

No.	Keyword	Results
#1	Sertraline OR Zoloft OR Altruline OR Lustral OR Apo-Sertraline OR Apo Sertraline OR Aremis OR Besitran OR Sealdin OR Gladem OR Novo-Sertraline OR Novo Sertraline OR ratio-Sertraline OR ratio Sertraline OR Rhoxal-sertraline OR Rhoxal sertraline OR Sertraline Hydrochloride OR Hydrochloride, Sertraline OR Sertraline Hydrochloride (1S-cis)-Isomer OR Gen-Sertraline OR Gen Sertraline	5877
#2	Fluoxetine OR Fluoxetin OR N-Methyl-gamma-(4-(trifluoromethyl)phenoxy)benzenepropanamine OR Lilly-110140 OR Lilly 110140 OR Lilly110140 OR Sarafem OR Fluoxetine Hydrochloride OR Prozac	15445
#3	Escitalopram OR Escitalopram Oxalate OR Lexapro	3114
#4	Citalopram OR Cytalopram OR Citalopram Hydrobromide OR Lu-10-171 OR Lu10171 OR Seropram OR Celexa	8614
#5	Paroxetine OR Paroxetine Hydrochloride Hemihydrate OR Paroxetine Hydrochloride, Hemihydrate OR Aropax OR BRL-29060 OR BRL 29060 OR BRL29060 OR FG-7051 OR FG 7051 OR FG7051 OR Paroxetine Acetate OR Paxil OR Seroxat OR Paroxetine Hydrochloride Anhydrous OR Paroxetine Maleate OR Paroxetine, cis-(+)-Isomer OR Paroxetine, cis-(-)-Isomer OR Paroxetine, trans-(+)-Isomer OR Paroxetine Hydrochloride	6738
#6	((((Sertraline OR Zoloft OR Altruline OR Lustral OR Apo-Sertraline OR Apo Sertraline OR Aremis OR Besitran OR Sealdin OR Gladem OR Novo-Sertraline OR Novo Sertraline OR ratio-Sertraline OR ratio Sertraline OR Rhoxal-sertraline OR Rhoxal sertraline OR Sertraline Hydrochloride OR Hydrochloride, Sertraline OR Sertraline Hydrochloride (1S-cis)-Isomer OR Gen-Sertraline OR Gen Sertraline) OR (Fluoxetine OR Fluoxetin OR N-Methyl-gamma-(4-(trifluoromethyl)phenoxy)benzenepropanamine OR Lilly-110140 OR Lilly 110140 OR Lilly110140 OR Sarafem OR Fluoxetine Hydrochloride OR Prozac)) OR (Escitalopram OR Escitalopram Oxalate OR Lexapro)) OR (Citalopram OR Cytalopram OR Citalopram Hydrobromide OR Lu-10-171 OR Lu10171 OR Seropram OR Celexa)) OR (Paroxetine OR Paroxetine Hydrochloride Hemihydrate OR Paroxetine Hydrochloride, Hemihydrate OR Aropax OR BRL-29060 OR BRL 29060 OR BRL29060 OR FG-7051 OR FG 7051 OR FG7051 OR Paroxetine Acetate OR Paxil OR Seroxat OR Paroxetine Hydrochloride Anhydrous OR Paroxetine Maleate OR Paroxetine, cis-(+)-Isomer OR Paroxetine, cis-(-)-Isomer OR Paroxetine, trans-(+)-Isomer OR Paroxetine Hydrochloride)	30396
#7	Forced swim test OR Porsolt	8536
#8	Rat OR Rattus OR Rattus norvegicus OR Rats, Norway OR Rats, Laboratory OR Laboratory Rat OR Laboratory Rats OR Rat, Laboratory	1841480
#9	((((((Sertraline OR Zoloft OR Altruline OR Lustral OR Apo-Sertraline OR Apo Sertraline OR Aremis OR Besitran OR Sealdin OR Gladem OR Novo-Sertraline OR Novo Sertraline OR ratio-Sertraline OR ratio Sertraline OR Rhoxal-sertraline OR Rhoxal sertraline OR Sertraline Hydrochloride OR Hydrochloride, Sertraline OR Sertraline Hydrochloride (1S-cis)-Isomer OR Gen-Sertraline OR Gen Sertraline) OR (Fluoxetine OR Fluoxetin OR N-Methyl-gamma-(4-(trifluoromethyl)phenoxy)benzenepropanamine OR Lilly-110140 OR Lilly 110140 OR Lilly110140 OR Sarafem OR Fluoxetine Hydrochloride OR Prozac)) OR (Escitalopram OR Escitalopram Oxalate OR Lexapro)) OR (Citalopram OR Cytalopram OR Citalopram Hydrobromide OR Lu-10-171 OR Lu10171 OR Seropram OR Celexa)) OR (Paroxetine OR	642

	Paroxetine Hydrochloride Hemihydrate OR Paroxetine Hydrochloride, Hemihydrate OR Aropax OR BRL-29060 OR BRL 29060 OR BRL29060 OR FG-7051 OR FG 7051 OR FG7051 OR Paroxetine Acetate OR Paxil OR Seroxat OR Paroxetine Hydrochloride Anhydrous OR Paroxetine Maleate OR Paroxetine, cis-(+)-Isomer OR Paroxetine, cis-(-)-Isomer OR Paroxetine, trans-(+)-Isomer OR Paroxetine Hydrochloride)) AND (forced swim test OR Porsolt)) AND (Rat OR Rattus OR Rattus norvegicus OR Rats, Norway OR Rats, Laboratory OR Laboratory Rat OR Laboratory Rats OR Rat, Laboratory)	

Table S2. EMBASE search result.

No.	Emtree keyword	Keywords	Results
#1	'sertraline'	1 methylamino 4 (3, 4 dichlorophenyl) tetralin OR 4 (3, 4 dichlorophenyl) 1, 2, 3, 4 tetrahydro n methyl 1 naphthalenamine OR 4 (3, 4 dichlorophenyl) 1, 2, 3, 4 tetrahydro n methylnaphthalen 1 amine OR 4 (3, 4 dichlorophenyl) n methyl tetralin 1 amine OR adjuvin OR altruline OR aremis OR atruline OR besitran OR cp 51974 OR cp 51974 01 OR cp 51974 1 OR cp 519741 OR cp51974 OR cp51974 01 OR cp51974 1 OR cp5197401 OR cp519741 OR dominum OR doxime OR fatral OR fridep OR gladem OR lesefer OR lustral OR n methyl 4 (3, 4 dichlorophenyl) 1, 2, 3, 4 tetrahydro 1 naphthylamine OR nudep OR seltra OR serad OR sercerin OR serlain OR serlift OR sertralin OR sertraline hydrochloride OR sertranex OR sertranquil OR sosser OR tatig OR tresleen OR zolof OR zoloft OR zosert	29 745
#2	'fluoxetine'	3 (4 trifluoromethylphenoxy) n methyl 3 phenylpropylamine OR 3 n methyl 3 phenyl 3 (4 trifluoromethylphenoxy) propylamine OR actan OR adofen OR afeksin OR alzac 20 OR andep OR andepin OR ansilan OR at 001 (serotonin uptake inhibitor) OR at001 (serotonin uptake inhibitor) OR atd 20 OR auroken OR auscap OR bioxetin OR captaton OR compound 110140 OR daforin OR dagrilan OR depren OR deprex (fluoxetine) OR deprex leciva OR deprexetin OR deprexin OR deprizac OR deproxin OR diesan OR digassim OR elizac OR exostrept OR felicium OR fldiss OR flotinal OR floxet OR fluctin OR fluctine OR fludac OR flufran OR fluketin OR flunil OR flunirin OR fluohexal OR fluoksetin OR fluoksetyna OR fluox OR fluox-puren OR fluoxac OR fluoxeren OR fluoxetin OR fluoxetina OR fluoxetine hydrochloride OR fluoxifar OR fluoxil OR fluoxone OR fluoxone divule OR fluoxtab OR fluronin OR flusac OR flustad OR flutin OR flutine OR flux (drug) OR fluxemed OR fluxen OR fluxet OR fluxetil OR fluxetin OR fluxil OR fluxomed OR fluzac OR fokeston OR fontex OR foxetin OR foxtin OR fropine OR fuloren OR gerozac OR her 801 OR her801 OR ladose OR lanclic OR lilly 110140 OR lilly110140 OR lorien OR lovan OR luramon OR ly 110140 OR ly110140 OR magrilan OR margrilan OR meropan OR modipran OR mutan OR n methyl 3 phenyl 3 (4 trifluoromethylphenoxy) propylamine OR n methyl 3 phenyl 3 [(alpha, alpha, alpha trifluoro para tolyl) oxy] propylamine OR n methyl 3 phenyl 3 [4 (trifluoromethyl) phenoxy] propan 1 amine OR nopres OR npl 2008 OR npl2008 OR nuzak OR olena OR oxactin OR oxedep OR phenylpropylamine, n methyl 3 (4 trifluoromethylphenoxy) OR plazeron OR plinzene OR portal (drug) OR pragmaten OR prizma OR proctin OR	52 410

		prodep OR prosac OR prozac OR prozac 20 OR prozac dispersible OR prozac weekly OR prozamel OR prozamin OR prozep OR prozit OR psipax OR qualisac OR rapiflux OR reconcile OR reneuron OR rowexetina OR salipax OR sanzur OR sarafem OR sartuzin OR selfemra OR serelsa OR seromex OR seronil OR sinzac OR sofelin OR stephadilat-s OR xeredien OR zactin OR zepax OR zinovat	
#3	'escitalopram'	cipralex OR enlift (drug) OR entact OR esciprex OR esciprex distab OR escitalopram oxalate OR lexapro OR lu 26054 0 OR lu 260540 OR lu260540 OR premalex OR prilect OR seroplex OR sipralexa OR zecidec OR zocital	15 037
#4	'citalopram'	1 (3 dimethylaminopropyl) 1 (4 fluorophenyl) 1, 3 dihydroisobenzofuran 5 carbonitrile OR 1 (3 dimethylaminopropyl) 1 (4 fluorophenyl) 5 phthalancarbonitrile OR 1 (3 dimethylaminopropyl) 1, 3 dihydro 1 (4 fluorophenyl) isobenzofuran 5 carbonitrile OR 5 phthalancarbonitrile, 1 (3 dimethylaminopropyl) 1 (4 fluorophenyl) OR acelopam OR adeprenal OR apo-cital OR aurex OR ceform OR celexa OR cilopress OR cinavol OR ciprager OR cipram OR cipramil OR cipraned OR ciprotan OR ciral OR citabax OR citacip OR citagen OR cital OR citalec OR citalich OR citalon OR citalonte OR citalopram hydrobromide OR citalopram hydrochloride OR citalopram ratiopharm OR citalostad OR citalox OR citalvir OR citapram OR citaxin OR citesint OR citopam OR citrol OR citronil OR cytalopram OR dalsan OR elopram OR exenadil OR frimaind OR futuril OR galopran OR humorap OR kaidor OR kitapram OR linisan OR lopracil OR lopraxer OR loxopram OR lu 10 171 OR lu 10171 OR lu10 171 OR lu10171 OR lupram OR malicon OR nitalapram OR oropram OR percitale OR pralotam OR pram (drug) OR pramital OR prefucet OR pricital OR prisdal OR psiconor OR recital (drug) OR renevil OR return (citalopram) OR ricap OR ropramin OR selon OR sepram OR seralgan OR seregra OR serital OR seropram OR seror OR sintopram OR sotovon OR talam OR talosin OR unstress (drug) OR varom OR vesema OR xadorek OR zanipram OR zd 211 OR zd211 OR zeclicid OR zentius OR zitolex OR zyloram	25 428
#5	'paroxetine'	3 (1, 3 benzodioxol 5 yloxymethyl) 4 (4 fluorophenyl) piperidine OR 4 (4 fluorophenyl) 3 [(3, 4 methylenedioxyphenoxy) methyl] piperidine OR arketis OR aropax OR aropax 20 OR aroxat OR brisdelle OR brl 29060 OR brl 29060a OR brl29060 OR brl29060a OR daparox OR deroxat OR dexorat OR divarius OR dropax OR dropaxin OR euplix OR eutimil OR fg 7051 OR fg7051 OR frosinor OR mesafem OR motivan OR nnc 207051 OR nnc207051 OR optipar OR paluxetil OR paluxon OR paroc OR parogen OR paroxedura OR paroxet OR paroxetin OR paroxetina OR paroxetine hydrochloride OR paroxetine mesilate OR paroxetine mesylate OR paroxia (drug) OR paxan OR paxil OR paxil cr OR paxtine OR paxxet OR pexeva OR serestill OR sereupin OR seroxat OR setine OR si 211103 OR si211103 OR solben (drug) OR syntopar OR tagonis	30 193
#6	'rat'	rats OR Rattus	2 095 898
#7	'forced swim test'	behavioural despair test OR experimental despair test OR forced swim (test) OR forced swim stress (test) OR forced swimming (test) OR forced swimming stress (test) OR forced swimming test OR Porsolt swim test OR Porsolt swimming test OR Porsolt test	14 833
#8	-	#1 OR #2 OR #3 OR #4 OR #5	94 166
#9	-	#1 OR #2 OR #3 OR #4 OR #5 AND #6 AND #7	1 145

Table S3. Web of Science search result.

No.	Keyword	Results
#1	Sertraline OR Zoloft OR Altruline OR Lustral OR Apo-Sertraline OR Apo Sertraline OR Aremis	7 946
#2	Fluoxetine OR Fluoxetin OR N-Methyl-gamma-(4-(trifluoromethyl)phenoxy)benzenepropanamine OR Lilly-110140 OR Lilly 110140 OR Lilly110140 OR Sarafem OR Fluoxetine Hydrochloride OR Prozac	22 297
#3	Escitalopram OR Escitalopram Oxalate OR Lexapro	4 365
#4	Citalopram OR Cytalopram OR Citalopram Hydrobromide OR Lu-10-171 OR Lu10171 OR Seropram OR Celexa	7 388
#5	Paroxetine OR Paroxetine Hydrochloride Hemihydrate OR Paroxetine Hydrochloride, Hemihydrate OR Aropax OR BRL-29060 OR BRL 29060 OR BRL29060 OR FG-7051 OR FG 7051 OR FG7051 OR Paroxetine Acetate OR Paxil OR Seroxat OR Paroxetine Hydrochloride Anhydrous OR Paroxetine Maleate OR Paroxetine, cis-(+)-Isomer OR Paroxetine, cis-(-)-Isomer OR Paroxetine, trans-(+)-Isomer OR Paroxetine Hydrochloride	9 282
#6	1 OR 2 OR 3 OR 4 OR 5	40 574
#7	Forced swim test	11 522
#8	Rat OR Rattus OR Rattus norvegicus OR Rats, Norway OR Rats, Laboratory OR Laboratory Rat OR Laboratory Rats OR Rat, Laboratory	1 997 081
#9	1 OR 2 OR 3 OR 4 OR 5 AND 7 AND 8	1 121

Table S4. Sensitivity analysis (SSRIs vs Control ADM)

Excluded studies	Frequency	Difference of means	SE	-95%CI	+95%CI	Z statistic	p-value	Remaining contribution	Precision change
Alam et al. 2018	571	-42.968086	5.182245	-53.125099	-32.811073	-8.291404	<0.000001	95.673%	-6.672%
Alshammari et al. 2019	571	-42.974036	5.560091	-53.871614	-32.076457	-7.729016	<0.000001	96.761%	0.133%
Ampuero et al. 2015 (Immobilisation + 0.7 mg)	570	-45.700585	5.600216	-56.676806	-34.724363	-8.160504	<0.000001	98.535%	0.856%
Ampuero et al. 2015 (Immobilisation + 3.5 mg)	568	-46.190757	5.622248	-57.21016	-35.171353	-8.21571	<0.000001	97.615%	1.252%
Ampuero et al. 2015 (RS + 0.7 mg)	570	-44.353811	5.617425	-55.363762	-33.343861	-7.895755	<0.000001	97.62%	1.166%
Ampuero et al. 2015 (RS + 3.5 mg)	568	-45.027996	5.62752	-56.057733	-33.99826	-8.001393	<0.000001	97.722%	1.347%
Berton et al. 1999	565	-45.689038	5.666927	-56.796011	-34.582065	-8.062401	<0.000001	96.73%	2.057%
Dashti et al. 2022	561	-46.151084	5.757093	-57.434779	-34.867389	-8.016387	<0.000001	95.767%	3.681%
Evans et al. 2012	517	-44.969672	6.144166	-57.012015	-32.927328	-7.319085	<0.000001	95.575%	10.652%
Guo et al. 2009	571	-45.131706	5.805962	-56.511183	-33.752228	-7.773337	<0.000001	95.753%	4.561%
Jagadeesan et al. 2019	571	-47.01895	5.445739	-57.692401	-36.345498	-8.634081	<0.000001	95.676%	-1.926%
Khedr et al. 2018(HFD)	559	-45.619627	5.716193	-56.823159	-34.416094	-7.980771	<0.000001	96.074%	2.944%
Khedr et al. 2018 (non-HFD)	560	-46.001496	5.721229	-57.214899	-34.788093	-8.040492	<0.000001	95.951%	3.035%

Lin et al. 2014	563	-44.8378	5.698508	-56.006671	-33.66893	-7.86834	<0.000001	96.16%	2.626%
Qi et al. 2018	571	-45.616911	5.622612	-56.637029	-34.596794	-8.113117	<0.000001	97.883%	1.259%
Ratajczak et al. 2019	571	-46.820421	5.568487	-57.734456	-35.906387	-8.408104	<0.000001	95.677%	0.284%
Szymańska et al. 2009	559	-46.705547	5.604385	-57.68994	-35.721154	-8.33375	<0.000001	95.654%	0.931%
Zavvari et al. 2020a	569	-44.822984	5.644956	-55.886895	-33.759072	-7.94036	<0.000001	97.166%	1.661%
Zavvari et al. 2020b	559	-44.463626	5.647811	-55.533132	-33.39412	-7.872718	<0.000001	96.864%	1.713%
Zhao et al. 2008	559	-45.802476	5.683334	-56.941606	-34.663345	-8.059085	<0.000001	96.402%	2.353%
Zhou et al. 2021	559	-44.153298	5.677748	-55.281479	-33.025117	-7.776552	<0.000001	95.69%	2.252%
Desbonnet et al. 2010	569	-46.335	5.794826	-57.692649	-34.97735	-7.995927	<0.000001	95.652%	4.36%
Hale et al. 2017	567	-46.331607	5.632176	-57.370469	-35.292745	-8.226236	<0.000001	97.223%	1.431%
Bah et al. 2011	570	-46.291124	5.689808	-57.442943	-35.139305	-8.135797	<0.000001	96.052%	2.469%
Marchetti et al. 2020	549	-44.62811	5.632307	-55.66723	-33.588991	-7.923593	<0.000001	97.402%	1.434%
Steyn et al. 2018	559	-46.254246	5.680217	-57.387267	-35.121224	-8.143041	<0.000001	96.208%	2.296%
Khedr et al. 2015	564	-44.491179	5.670374	-55.604907	-33.377451	-7.846252	<0.000001	96.373%	2.119%
Zangen et al. 2001	559	-44.873944	5.640462	-55.929047	-33.818842	-7.955721	<0.000001	97.307%	1.58%
Solak et al. 2022	555	-44.38774	5.646448	-55.454576	-33.320904	-7.861179	<0.000001	96.833%	1.688%

Table S5 Sensitivity analysis (Control ADM vs Control)

Excluded studies	Frequency	Difference of means	SE	-95%CI	+95%CI	Z statistic	p-value	Remaining contribution	Precision change
Alam et al. 2018	527	47.187925	7.622515	32.24807	62.127781	6.190598	<0.000001	95.711%	-0.286%
Dashti et al. 2022	517	49.234115	8.042737	33.47064	64.997589	6.121562	<0.000001	95.752%	5.211%
Guo et al. 2009	527	49.572696	8.053773	33.787591	65.3578	6.155214	<0.000001	95.743%	5.355%
Jagadeesan et al. 2019	527	51.611739	7.307566	37.289172	65.934306	7.062781	<0.000001	95.692%	-4.406%
Khedr et al. 2015	520	48.991209	7.86641	33.57333	64.409089	6.2279	<0.000001	96.042%	2.904%
Qi et al. 2018	527	47.870113	7.753783	32.672977	63.067248	6.173775	<0.000001	97.127%	1.431%
Solak et al. 2022	511	47.676526	7.784186	32.419802	62.933251	6.124793	<0.000001	96.386%	1.829%
Zavvari et al. 2020a	525	48.058712	7.806939	32.757394	63.360031	6.155897	<0.000001	96.295%	2.126%
Zavvari et al. 2020b	515	47.58818	7.79069	32.318708	62.857653	6.108339	<0.000001	96.172%	1.914%
Zhou et al. 2021	515	47.419149	7.748607	32.23216	62.606139	6.1197	<0.000001	95.723%	1.363%
Zangen et al. 2001	515	48.473168	7.798164	33.189047	63.757288	6.215972	<0.000001	96.59%	2.012%
Ampuero et al. 2015 (Immobilisation)	516	50.433027	7.774729	35.194838	65.671216	6.486789	<0.000001	96.914%	1.705%
Desbonnet et al. 2010	521	51.228566	7.750248	36.038358	66.418774	6.609926	<0.000001	95.79%	1.385%
Marchetti et al. 2020	511	49.436387	7.787152	34.173851	64.698924	6.348456	<0.000001	96.902%	1.867%
Bah et al. 2011	527	50.904325	7.810624	35.595783	66.212867	6.517319	<0.000001	95.943%	2.175%
Lin et al. 2014	524	49.897428	7.842813	34.525796	65.269059	6.362185	<0.000001	96.149%	2.596%
Ratajczak et al. 2019	527	51.779262	7.618814	36.84666	66.711864	6.796236	<0.000001	95.808%	-0.335%
Szymańska et al. 2009	515	50.649978	7.946087	35.075934	66.224022	6.374204	<0.000001	95.681%	3.947%
Berton et al. 1999	521	52.227578	7.703056	37.129865	67.325291	6.780111	<0.000001	96.566%	0.767%
Alshammari et al. 2019	527	48.32895	7.813626	33.014525	63.643375	6.185214	<0.000001	96.331%	2.214%
Evans et al. 2012	473	48.161453	7.961244	32.557703	63.765204	6.049489	<0.000001	95.646%	4.145%
Ampuero et al. 2015 (RS)	516	49.786182	7.842576	34.415014	65.157349	6.348192	<0.000001	96.169%	2.593%
Khedr et al. 2018 (HFD)	515	51.136278	7.784901	35.878153	66.394403	6.568649	<0.000001	95.945%	1.838%
Khedr et al. 2018 (non-HFD)	518	50.354031	7.896476	34.877223	65.830839	6.376773	<0.000001	95.822%	3.298%
Zhao et al. 2008	515	49.20653	7.84982	33.821166	64.591895	6.268491	<0.000001	96.149%	2.687%

Table S6. Publication bias Egger's test.

Asymmetry analysis	SSRIs vs Control (ADM)	Control (ADM) vs Control
b Egger coefficient	0.272883	-1.822306
SE(b)	1.179183	1.675862
-95% CI for b coefficient	-2.1466	-5.281114
+95% CI for b coefficient	2.692367	1.636503
t-test statistic for b	0.231417	-1.087384
Degrees of freedom	27	24
p-value	0.818736	0.287667

Table S7. Comprehensive list of included studies.

**Author, Year**<br><br>**Country**<br><br>**Journal (Publisher)**<br><br>**Funding**<br><br>**Conflict of interest (COI)**	**Animal model of depression**<br><br>**Study arms (groups)**	**Rattus norvegicus strain**<br><br>**During FST:**<br><br>**Number of animals**<br><br>**Developmental stage and gender**<br><br>**Animal weight (g)**	**Investigated drugs (dose, route of administration)**<br><br>**Duration of drug administration (days)**	**FST results (mean ± SD)**
Alam et al. 2018<br><br>India<br><br>Artif Cells Nanomed Biotechnol (Taylor&Francis Online)<br><br>NR<br><br>COI: None	Chronic Unpredictable Mild Stress (CUMS)<br><br>G1: Control<br>G2: CUMS Control<br>G3: CUMS Fluoxetine	Albino Wistar<br><br>G1: 6<br>G2: 6<br>G3: 6<br><br>Adults, Males<br><br>150-200 g	G1: Saline<br>G2: None<br>G3: Fluoxetine 10 mg/kg *p.o.*<br><br>28 days	G1: 34.66 ± 10.63<br>G2: 137.33 ± 6.58<br>G3: 46.5 ± 8.62
Alshammari et al. 2019<br><br>Saudi Arabia<br><br>Plos ONE (Public Library of Science)<br><br>Government<br><br>COI: None	Social Isolation (SI)<br><br>G1: Control<br>G2: SI Control<br>G3: SI Fluoxetine	Wistar<br><br>G1: 6<br>G2: 6<br>G3: 6<br><br>Adults, Males<br><br>150-170 g	G1: None<br>G2: None<br>G3: Fluoxetine 25 mg/kg *p.o.*<br><br>7 days	G1: 75.00 ± 4.45<br>G2: 157.50 ± 38.94<br>G3: 42.50 ± 6.12
Ampuero et al. 2015	Restraint stress (RS)	Sprague – Dawley	G1: Saline<br>G2: Saline	G1: 174.10 ± 23.10<br>G2: 218.60 ± 35.00

**Author, Year**<br><br>**Country**<br><br>**Journal (Publisher)**<br><br>**Funding**<br><br>**Conflict of interest (COI)**	**Animal model of depression**<br><br>**Study arms (groups)**	**Rattus norvegicus strain**<br><br>**During FST:**<br><br>**Number of animals**<br><br>**Developmental stage and gender**<br><br>**Animal weight (g)**	**Investigated drugs (dose, route of administration)**<br><br>**Duration of drug administration (days)**	**FST results (mean ± SD)**
Sweden<br><br>Int J Neuropsychoph<br>(Oxford Acadmic)<br><br>Academic<br><br>COI: None	G1: Control<br>G2: RS Control<br>G3: RS Fluoxetine 0.7 mg<br>G4: RS Fluoxetine 3.5 mg	G1: 5<br>G2: 8<br>G3: 5<br>G4: 7<br><br>Adults, Males<br><br>250-280 g	G3: Fluoxetine 0.7 mg *i.p.*<br>G4: Fluoxetine 3.5 mg *i.p.*<br><br>28 days	G3: 133.20 ± 47.40<br>G4: 160.10 ± 59.60
Ampuero et al. 2015<br><br>Sweden<br><br>Int J Neuropsychoph (Oxford Acadmic)<br><br>Academic<br><br>COI: None	Immobilisation<br><br>G1: Control<br>G2: Immobilisation Control<br>G3: Immobilisation Fluoxetine 0.7 mg<br>G4: Immobilisation Fluoxetine 3.5 mg	Sprague – Dawley<br><br>G1: 5<br>G2: 8<br>G3: 5<br>G4: 7<br><br>Adults, Males<br><br>250-280 g	G1: Saline<br>G2: Saline<br>G3: Fluoxetine 0.7 mg *i.p.*<br>G4: Fluoxetine 3.5 mg *i.p.*<br><br>28 days	G1: 174.10 ± 23.10<br>G2: 197.00 ± 64.60<br>G3: 176.30 ± 66.70<br>G4: 186.80 ± 23.40
Bah et al. 2011<br><br>Taiwan,<br><br>Behav Brain Res (ELSEVIER)<br><br>Industry<br><br>COI: None	Myocardial infarction<br><br>G1: Control<br>G2: Myocardial infarction Control<br>G3: Myocardial infarction Escitalopram	Sprague-Dawley<br><br>G1: 6<br>G2: 6<br>G3: 7<br><br>Adults, Males<br><br>350–375 g	G1: Saline<br>G2: Saline<br>G3: Escitalopram 10 mg/kg *i.p.*<br><br>14 days	G1: 34.00 ± 12.44<br>G2: 52.33 + 21.65<br>G3: 30.14 ± 8.36
Berton et al. 1999<br><br>France<br><br>Neurosci (ELSEVIER)<br><br>Government<br><br>COI: NR	Social defeat<br><br>G1: Control<br>G2: Social defeat Control<br>G3: Social defeat Fluoxetine	Lewis<br><br>G1: 9<br>G2: 9<br>G3: 9<br><br>Adults, Males<br><br>210-230 g	G1: Distilled water<br>G2: Distilled water<br>G3: Fluoxetine 7.5 mg/kg *i.p.*<br><br>21 days	G1: 205.00 ± 42.00<br>G2: 180.00 ± 39.00<br>G3: 145.00 ± 27.00
Dashti et al. 2022<br><br>Iran<br><br>J Chem Neuroanat (ELSEVIER)	Chronic Unpredictable Mild Stress (CUMS)<br><br>G1: Control<br>G2: CUMS Contol	Wistar<br><br>G1: 11<br>G2: 11	G1: Saline<br>G2: Saline<br>G2: Fluoxetine 20mg/kg *p.o.*<br><br>21 days	G1: 46.14 ± 13.49<br>G2: 103.71 ± 15.75<br>G3: 76.29 ± 11.90

Author, Year<br><br>Country<br><br>Journal (Publisher)<br><br>Funding<br><br>Conflict of interest (COI)	Animal model of depression<br><br>Study arms (groups)	Rattus norvegicus strain<br><br>During FST:<br><br>Number of animals<br><br>Developmental stage and gender<br><br>Animal weight (g)	Investigated drugs (dose, route of administration)<br><br>Duration of drug administration (days)	FST results (mean ± SD)
Academic, Foundation<br><br>COI: None	G3: CUMS Fluoxetine	G3: 11<br><br>Adults, Males<br><br>220–250 g		
Desbonnet et al. 2010<br><br>Ireland<br><br>Neuroscience (ELSEVIER)<br><br>Academic<br><br>COI: NR	Maternal Deprivation (MD)<br><br>G1: Control<br>G2: MD Control<br>G3: MD Citalopram	Sprague-Dawley<br><br>G1: 11<br>G2: 7<br>G3: 7<br><br>Adults, Males<br><br>150-200 g	G1: None<br>G2: None<br>G3: Citalopram 30 mg/kg p.o.<br><br>40 days	G1: 18.70 ± 21.23<br>G2: 30.86 ± 8.62<br>G3: 6.80 ± 6.27
Evans et al. 2012<br><br>New Zealand<br><br>Neuropharmacology (ELSEVIER)<br><br>Academic, Government<br><br>COI: None	Social Isolation (SI)<br><br>G1: Control<br>G2: SI Control<br>G3: SI Fluoxetine	Wistar<br><br>G1:33<br>G2:33<br>G3: 33<br><br>Adults, Males<br><br>NR	G1: Placebo pellet<br>G2: Placebo pellet<br>G3: Fluoxetine 10 mg/kg p.o.<br><br>28 days	G1: 44.00 ± 11.00<br>G2: 125.00 ± 8.00<br>G3: 68.00 ± 8.00
Guo et al. 2009<br><br>China<br><br>Prog Neuro-Psychopharmacol Biol Psychiatry (ELSEVIER)<br><br>Government<br><br>COI: None	Chronic Unpredictable Mild Stress (CUMS)<br><br>G1:Control<br>G2:MCAO^x CUMS Control<br>G3: MCAO^x CUMS Fluoxetine<br><br>^{x} MCAO – middle cerebral artery occlusion	Sprague – Dawley<br><br>G1: 6<br>G2: 6<br>G3: 6<br><br>Adults, Males<br><br>210–250 g	G1: None<br>G2: None<br>G3: Fluoxetine 5mg/kg *i.p.*<br><br>18 days	G1: 43.8 ± 8.8<br>G2: 93.7 ±11.8<br>G3: 42.7 ± 7.7
Hale et al. 2017<br><br>USA<br><br>Prog Neuro-Psychopharmacol Biol Psychiatry (ELSEVIER)<br><br>Academic, Government, Foundation	Hyperthermia (thermoregulation 23^{0}C vs 37^{0}C)<br><br>G1: Control<br>G2: Hyperthermia Control<br>G3: Hyperthermia Citalopram	Wistar<br><br>G1: 8<br>G2: 8<br>G3: 8<br><br>Adolescents, males	G1: saline<br>G2: saline<br>G3: citalopram 5mg/kg *s.c.*	G1: 153.86 ± 58.60<br>G2: 127.76 ± 34,67<br>G3: 117.44 ± 46.24

**Author, Year**<br><br>**Country**<br><br>**Journal (Publisher)**<br><br>**Funding**<br><br>**Conflict of interest (COI)**	**Animal model of depression**<br><br>**Study arms (groups)**	**Rattus norvegicus strain**<br><br>**During FST:**<br><br>**Number of animals**<br><br>**Developmental stage and gender**<br><br>**Animal weight (g)**	**Investigated drugs (dose, route of administration)**<br><br>**Duration of drug administration (days)**	**FST results (mean ± SD)**
COI: None		145 g		
Jagadeesan et al. 2019<br><br>Malaysia<br><br>Biomed Res Ther (BIOMEDPRESS)<br><br>Academic<br><br>COI: None	Chronic Unpredictable Mild Stress (CUMS)<br><br>G1: Control<br>G2: CUMS Control<br>G3: CUMS Fluoxetine	Wistar<br><br>G1: 6<br>G2: 6<br>G3: 6<br><br>Adults, Males<br><br>180-220 g	G1: Saline<br>G2: Saline<br>G3: Fluoxetine 10 mg/kg *i.p.*<br><br>56 days	G1: 5.00 ± 1.40<br>G2: 10.00 ± 10.83<br>G3: 3.50 ± 1.70
Khedr et al. 2015<br><br>Egypt<br><br>Neuropsychiatr Dis Treat (DOVE MEDICAL PRESS)<br><br>NR<br><br>COI: None	Chronic Unpredictable Mild Stress (CUMS)<br><br>G1: Control<br>G2: CUMS Control<br>G3: CUMS Paroxetine	Wistar<br><br>G1: 10<br>G2: 9<br>G3: 10<br><br>NR, Males<br><br>200 ± 50 g	G1: Saline (and dimethylsulfoxide)<br>G2: Saline (and dimethylsulfoxide)<br>G3: Paroxetine 10 mg/kg *i.p.*<br><br>28 days	G1: 57.43 ± 22.98<br>G2: 121.4 ± 27.99<br>G3: 53.6 ± 26.46
Khedr et al. 2018<br><br>Egypt<br><br>Naunyn-Schmiedebergs Arch Pharmacol (SPRINGER)<br><br>NR<br><br>COI: None	Restraint stress (RS) (non-high fat diet)<br><br>G1: RS Control<br>G2: RS Control<br>G3: RS Fluoxetine	Wistar<br><br>G1: 10<br>G2: 11<br>G3: 12<br><br>NR, Males<br><br>150-200 g	G1: None<br>G2: Vehicle<br>G3: Fluoxetine 10mg/kg *p.o.*<br><br>28 days	G1: 51.4 ± 9.39<br>G2: 83.3 ± 24.21<br>G3: 53.4 ± 13.51
Khedr et al. 2018<br><br>Egypt<br><br>Naunyn-Schmiedebergs Arch Pharmacol (SPRINGER)<br><br>NR<br><br>COI: None	Restraint stress (RS) (high fat diet)<br><br>G1: RS Control<br>G2: RS Control<br>G3: RS Fluoxetine	Wistar<br><br>G1: 12<br>G2: 12<br>G3: 12<br><br>NR, Males<br><br>150-200 g	G1: None<br>G2: Vehicle<br>G3: Fluoxetine 10mg/kg *p.o.*<br><br>28 days	G1: 79.25 ± 16.25<br>G2: 92.08 ± 31.45<br>G3: 53.33 ± 10.98

Author, Year<br><br>Country<br><br>Journal (Publisher)<br><br>Funding<br><br>Conflict of interest (COI)	Animal model of depression<br><br>Study arms (groups)	Rattus norvegicus strain<br><br>During FST:<br><br>Number of animals<br><br>Developmental stage and gender<br><br>Animal weight (g)	Investigated drugs (dose, route of administration)<br><br>Duration of drug administration (days)	FST results (mean ± SD)
Lin et al. 2014<br><br>Taiwan<br><br>Behav Brain Res (ELSEVIER)<br><br>Government<br><br>COI: None	Low-dose lipopolysaccharide (LPS)<br><br>G1: Control<br>G2: LPS Control<br>G3: LPS Fluoxetine	Sprague-Dawley<br><br>G1: 7<br>G2: 8<br>G3: 12<br><br>Adults, Male<br><br>NR	G1: Saline (phosphate-buffered)<br>G2: Saline (phosphate-buffered)<br>G3: Fluoxetine 10mg/kg *i.p.*<br><br>28 days	G1: 88.30 ± 31.43<br>G2: 130.05 ± 17.81<br>G3: 72.00 ± 29.06
Marchetti et al. 2020<br><br>Italy<br><br>Eur Neuropsychopharmacol (ELSEVIER)<br><br>Academic, Government<br><br>COI: None	Maternal deprivation (MD)<br><br>G1: Control<br>G2: MD Control<br>G3: MD Escitalopram	Flinders sensitive line<br><br>G1: 10<br>G2: 18<br>G3: 16<br><br>Adults, Males<br><br>NR	G1: Vehicle<br>G2: Vehicle<br>G3: Escitilopram 25 mg/kg *p.o.*<br><br>21 days	G1: 147.6 ± 49.74<br>G2: 201.77 ± 73.77<br>G3: 130.00 ± 55.36
Qi et al. 2018<br><br>China<br><br>ACS Chem Neurosci (ASC PUBLICATIONS)<br><br>Academic, Government, Foundation<br><br>COI: None	Chronic Unpredictable Mild Stress (CUMS)<br><br>G1: Control<br>G2: CUMS Control<br>G3: CUMS Fluoxetine	Sprague-Dawley<br><br>G1: 6<br>G2: 6<br>G3: 6<br><br>Adults, Males<br><br>210-230 g	G1: Saline<br>G2: Saline<br>G3: Fluoxetine 10 mg/kg *i.p.*<br><br>28 days	G1: 31.83 ± 10.97<br>G2: 139.33 ± 63.76<br>G3: 107.00 ± 22.70
Ratajczak et al. 2019<br><br>Poland<br>Acta Neurobiol Exp (Wars) (NENCKI INSTITUTE of EXPERIMENTAL BIOLOGY)<br><br>NR<br><br>COI: NR	Prenatal stress (PS)<br><br>G1: Control<br>G2: PS Control<br>G3: PS Fluoxetine	Wistar<br><br>G1: 6<br>G2: 6<br>G3: 6<br><br>NR, Males<br><br>NR	G1: Saline<br>G2: Saline<br>G3: Fluoxetine 5mg/kg *i.p.*<br><br>21 days	G1: 283.16 ± 17.41<br>G2: 282.66 ± 6.51<br>G3: 271.00 ± 8.91
Solak et al. 2022<br><br>Turkey	Chronic Unpredictable Mild Stress (CUMS)<br><br>G1: Control	Albino Wistar<br><br>G1: 14<br>G2: 14	G1: Dimethyl sulfoxide *s.c.*<br>G2: Dimethyl sulfoxide *s.c.*<br>G3: Setraline 10mg/kg *s.c.*	G1: 49.07 ±42.58<br>G2: 149.5 ± 45.98<br>G3: 75.28 ± 42.58

Author, Year<br><br>Country<br><br>Journal (Publisher)<br><br>Funding<br><br>Conflict of interest (COI)	Animal model of depression<br><br>Study arms (groups)	Rattus norvegicus strain<br><br>During FST:<br><br>Number of animals<br><br>Developmental stage and gender<br><br>Animal weight (g)	Investigated drugs (dose, route of administration)<br><br>Duration of drug administration (days)	FST results (mean ± SD)
Neurochem Res (SPRINGER)<br><br>Academic<br><br>COI: None	G2: CUMS Control<br>G3: CUMS Sertraline	G3: 14<br><br>Adults, Males<br><br>300-400 g*	15 days	
Steyn et al. 2018<br><br>South Africa<br><br>Beahv Brain Res (ELSEVIER)<br><br>Government, Foundation, Industry<br><br>COI: None	Flinders sensitive line (FSL)<br><br>G1: None<br>G2: FSL Control<br>G3: FSL Escitalopram	Flinders sensitive line<br><br>G2:12<br>G3: 12<br><br>Adolescents, Males<br><br>NR	G2: Saline<br>G3: Escitalopram 10 mg/kg *s.c.*<br><br>14 days	G2: 185.7 ± 27.36<br>G3: 163.6 ± 26.32
Szymańska et al. 2009<br><br>Poland<br><br>Psychoneuroendocrinology (ELSEVIER)<br><br>Government<br><br>COI: None	Prenatal stress (PS)<br><br>G1: Control<br>G2: PS Control<br>G3: PS Fluoxetine	Sprague – Dawley<br><br>G1: 12<br>G2: 12<br>G3: 12<br><br>Adults, Males<br><br>200—250 g	G1: Saline<br>G2: Saline<br>G3: Fluoxetine 10 mg/kg *i.p.*<br><br>21 days	G1: 262.40 ± 12.47<br>G2: 288.30 ± 6.58<br>G3: 273.70 ± 12.47
Zangen et al. 2001<br><br>US<br><br>Psychopharmacology (SPRINGER)<br><br>Government<br><br>COI: NR	Flinders sensitive line (FSL)<br><br>G1: Control<br>G2: FSL Control<br>G3: FSL Paroxetine	Sprague-Dawley<br><br>G1: 12<br>G2: 12<br>G3: 12<br><br>NR, Males<br><br>230–260 g	G1: Saline<br>G2: Saline<br>G3: Paroxetine 7.5 mg/kg *i.p.*<br><br>18 days	G1: 113.00 ± 38.10<br>G2: 194.00 ± 55.42<br>G3: 132.00 ± 48.49
Zavvarii et all. 2020 a<br><br>Iran<br><br>J Chem Neuroanat (ELSEVIER)<br><br>Academic<br><br>COI: None	Chronic Unpredictable Mild Stress (CUMS)<br><br>G1: Control<br>G2: CUMS Control<br>G3: CUMS Fluoxetine	Wistar<br><br>G1: 7<br>G2: 7<br>G3: 7<br><br>Adults, Males<br><br>200-250 g	G1: None<br>G2: None<br>G3: Fluoxetine 18mg/kg *p.o.*<br><br>24 days	G1: 43.20 ± 15.34<br>G2: 132.40 ± 38.09<br>G3: 69.50 ± 36.24

**Author, Year**<br><br>**Country**<br><br>**Journal (Publisher)**<br><br>**Funding**<br><br>**Conflict of interest (COI)**	**Animal model of depression**<br><br>**Study arms (groups)**	**Rattus norvegicus strain**<br><br>**During FST:**<br><br>**Number of animals**<br><br>**Developmental stage and gender**<br><br>**Animal weight (g)**	**Investigated drugs (dose, route of administration)**<br><br>**Duration of drug administration (days)**	**FST results (mean ± SD)**
Zavvari et al. 2020 b<br><br>Iran<br><br>J Chem Neuroanat (ELSEVIER)<br><br>Academic<br><br>COI: None	Chronic Unpredictable Mild Stress (CUMS)<br><br>G1: Control<br>G2: CUMS Control<br>G3: CUMS Fluoxetine	Wistar<br><br>G1: 12<br>G2: 12<br>G3: 12<br><br>Adults, Males<br><br>200–250 g	G1: None<br>G2: None<br>G3: Fluoxetine 18 mg/kg *p.o.*<br><br>24 days	G1: 39.30 ± 23.55<br>G2:139.00 ± 41.56<br>G3: 66.80 ± 41.91
Zhao et al. 2008<br><br>China<br><br>Behav Brain Res (ELSEVIER)<br><br>Foundation, Academic<br><br>COI: NR	Restraint stress (RS)<br><br>G1: Control<br>G2: RS Control<br>G3: RS Fluoxetine	Sprague–Dawley<br><br>G1: 12<br>G2: 12<br>G3: 12<br><br>NR, Males<br><br>180–220 g	G1: None<br>G3: Pure water<br>G4: Fluoxetine 5 ml/kg *p.o.*<br><br>21 days	G1: 37.00 ± 31.17<br>G2: 96.00 ± 34.64<br>G3: 63.00 ± 27.71
Zhou et al. 2021<br><br>China<br><br>Front Pharmacol (FRONTIERS)<br><br>Government<br><br>COI: None	Chronic Unpredictable Mild Stress (CUMS)<br><br>G1: Control<br>G2: CUMS Control<br>G3: CUMS Fluoxetine	Sprague – Dawley<br><br>G1: 12<br>G2: 12<br>G3: 12<br><br>Adolescents, Males<br><br>180–220 g	G1: None<br>G2: Distilled water<br>G3: Fluoxetine 2mg/kg *p.o.*<br><br>21 days	G1: 115.80 ± 13.80<br>G2: 213.70 ± 12.90<br>G3: 142.20 ± 10.10
